# A multispecies dependent double‐observer model: A new method for estimating multispecies abundance

**DOI:** 10.1002/ece3.2946

**Published:** 2017-04-04

**Authors:** Jessie D. Golding, J. Joshua Nowak, Victoria J. Dreitz

**Affiliations:** ^1^ Avian Science CenterWildlife Biology ProgramUniversity of MontanaMissoulaMTUSA; ^2^ Wildlife Biology ProgramUniversity of MontanaMissoulaMTUSA

**Keywords:** abundance model, Bayesian N‐mixture model, dependent double‐observer, false positive, multiple species

## Abstract

Conservation of biological communities requires accurate estimates of abundance for multiple species. Recent advances in estimating abundance of multiple species, such as Bayesian multispecies N‐mixture models, account for multiple sources of variation, including detection error. However, false‐positive errors (misidentification or double counts), which are prevalent in multispecies data sets, remain largely unaddressed. The dependent‐double observer (DDO) method is an emerging method that both accounts for detection error and is suggested to reduce the occurrence of false positives because it relies on two observers working collaboratively to identify individuals. To date, the DDO method has not been combined with advantages of multispecies N‐mixture models. Here, we derive an extension of a multispecies N‐mixture model using the DDO survey method to create a multispecies dependent double‐observer abundance model (MDAM). The MDAM uses a hierarchical framework to account for biological and observational processes in a statistically consistent framework while using the accurate observation data from the DDO survey method. We demonstrate that the MDAM accurately estimates abundance of multiple species with simulated and real multispecies data sets. Simulations showed that the model provides both precise and accurate abundance estimates, with average credible interval coverage across 100 repeated simulations of 94.5% for abundance estimates and 92.5% for detection estimates. In addition, 92.2% of abundance estimates had a mean absolute percent error between 0% and 20%, with a mean of 7.7%. We present the MDAM as an important step forward in expanding the applicability of the DDO method to a multispecies setting. Previous implementation of the DDO method suggests the MDAM can be applied to a broad array of biological communities. We suggest that researchers interested in assessing biological communities consider the MDAM as a tool for deriving accurate, multispecies abundance estimates.

## 1 Introduction

Effective conservation of biodiversity, the abundance of individuals and species within a given area, requires reliable models to predict changes in the abundance of multiple species. Species have different life history strategies and often respond differently to natural and anthropogenic disturbances (Buckland, Magurran, Green, & Fewster, 2005; Tulloch, Possingham, & Wilson, 2010; Tylianakis, Didham, Bascompte, & Wardle, 2008; Wiens, Hayward, Holthausen, & Wisdom, 2008). The underlying cause of changes in biodiversity may be complex. For example, abundance of one species may vary in response to changes in abiotic conditions. This can lead to changes in other species abundance through other biotic interactions. Multispecies abundance information can help disentangle these complex responses (Barnagaud, Barbaro, Papaix, Deconchat, & Brockerhoff, 2014; Dorazio & Connor, 2014; Ockendon et al., 2014). Empirical evidence suggests that if the multiple species are selected based on similar life history traits (i.e., they are limited by the same biological processes), they can represent what is occurring in the community (Lindenmayer et al., 2014).

However, many multispecies studies fail to account for imperfect detection (Iknayan, Tingley, Furnas, & Beissinger, 2014), one of the main challenges associated with any abundance estimates (Schwarz & Seber, 1999; Seber, 1986, 1992). Accounting for imperfect detection is important when considering a wide variety of species with different detection rates. Imperfect detection results from two processes governing the components of detection (Table 1): (1) Biological processes that influence abundance and determine availability; and (2) observation processes that determine detectability, which can be affected by species, observer experience, time of day, and other factors (Alldredge, Simons, & Polluck, 2007; Farnsworth et al., 2002; Pacifici, Simons, & Pollock, 2008; Simons, Alldredge, Pollock, & Wettroth, 2007). Failing to account for imperfect detection when monitoring multiple species can lead to incorrect inferences about drivers of change in abundance or biodiversity (Buckland et al., 2005; Iknayan et al., 2014; Kéry & Schaub, 2012). Imperfect detection from different sources can produce similar abundance patterns that result from entirely different mechanisms. For example, a common species may be consistently available (i.e., present), but have a low detectability because of cryptic behavior. A rare species, on the other hand, may be mostly unavailable across sites (i.e., present only in a low density), but have high detectability as a result of conspicuous vocalization. All of these factors can differentially affect the observation of each species, producing different observed counts, and ultimately abundance estimates.

**Table 1:** Two processes, biological and observation, influence the two components of detection, availability, and detectability. Detection error results from two specific combinations of these two processes

Biological process	Observation process	Detection error present?	Detection outcome
Availability^a^	Detectability^b^		
Available (present)	Detected	No	True positive
	Not detected	Yes	False negative
Not available (not present)	Detected	Yes	False positive
	Not detected	No	True negative

a The probability that an individual is present and available for observation at a plot. Independent of detectability.

b The probability an observer detects an individual. Dependent on availability.

Recent abundance estimation methods based on N‐mixture models have expanded from a single species (Royle, 2004) to a multispecies context (Kéry, Royle, & Schmid, 2005). N‐mixture models produce adjusted abundance estimates using information contained within repeated counts to estimate detection. The extension of an N‐mixture structure from single species abundance model to multispecies abundance models allows simultaneous estimates of abundance and detection probability for numerous species using spatially and temporally replicated counts (Kéry & Schaub, 2012). Multispecies N‐mixture models can incorporate species‐specific or site‐specific covariates, such as habitat type, as well as share information between species in both the biological and observation processes (Chandler et al., 2013; Dorazio & Connor, 2014; Iknayan et al., 2014; Yamaura et al., 2012; Yamaura et al., 2016). As a result, multispecies N‐mixture models are being used to assess various components of biodiversity, including the response of forest bird biodiversity to different types of land‐use practices (Chandler et al., 2013; Yamaura et al., 2012), community assembly of forest birds (Barnagaud et al., 2014), species interactions (Dorazio & Connor, 2014), and to examine species‐area relationships (Yamaura et al., 2016).

One of the major limitations of current multispecies N‐mixture models is that they do not address imperfect detection in the form of false‐positive errors (i.e., the detection of an individual that is not present because of either misidentification or double count of another individual; hereafter “false positives”) (Iknayan et al., 2014). False positives have been documented in many different types of ecological survey data (Miller et al., 2015). Royle and Link (2006) suggested that false positives due to misidentification can be highly prevalent in multispecies data. If not accounted for, even small rates of false positives can lead to substantial biases (Connors, Cooper, Peterman, & Dulvy, 2014; Fitzpatrick, Preisser, Ellison, & Elkinton, 2009; Miller et al., 2011; Royle & Link, 2006). Design‐based (e.g., Miller et al., 2012; Molinari‐Jobin et al., 2012) and statistical methods (e.g., Royle & Link, 2006) have been developed to account for sources of variation that lead to false positives, but they are difficult to incorporate. Most often, researchers assume that false positives do not occur (Miller et al., 2012; Nichols et al., 2000; Royle & Link, 2006).

The dependent double‐observer (DDO) method is a survey method that reduces false‐positive observations using removal‐based methodology to calculate detectability (Nichols et al., 2000). The method uses two observers with different roles. The primary observer dictates all individuals he/she observes during a survey to the secondary observer. The secondary observer notes the identity and location of the individuals observed by the primary observer. In addition, the secondary observer notes individuals missed by the primary observer. This process relies on the secondary observer verifying the observations of the primary observer, making an incorrect detection in the majority of the observations less likely than with a single observer acting alone (Nichols et al., 2000). In addition, the DDO has been shown to reduce false negatives (i.e., missed detections) compared to single observer methods (Golding & Dreitz, 2016; Kissling & Garton, 2006). The DDO method has been successfully applied in arid and woodland environments to estimate avian abundance (Kissling & Garton, 2006; Nichols et al., 2000; Tipton, Doherty, & Dreitz, 2009) and occupancy (Tipton, Dreitz, & Doherty, 2008).

Although recent studies have used multispecies N‐mixture models to track biodiversity in response to different land use types (Chandler et al., 2013; Yamaura et al., 2012), none have provided methods to reduce false positives. Here, we provide an expansion of the multispecies N‐mixture framework to account for false positives by incorporating the DDO method in a multispecies, multiseason framework. We simulated abundance and count data for four species to develop this multispecies dependent double‐observer abundance model (MDAM). We then applied the MDAM to case study data collected on prairie songbirds over multiple years on private and public lands in eastern Montana. Songbirds are becoming increasingly important indicators in diversity monitoring (Iknayan et al., 2014). Studies have shown that changes in songbird abundance and biodiversity are reliable indicators of impacts resulting from anthropogenic disturbance and land management in numerous ecosystems (e.g., Bradford et al., 1998; Canterbury, Martin, Petit, Petit, & Bradford, 2000; Coppedge, Engle, Masters, & Gregory, 2006; Coppedge, Fuhlendorf, Harrell, & Engle, 2008; Mac Nally, 1997; Schulze et al., 2004). With the reduction in false positives, the MDAM can provide more reliable estimates and rigorous inference about changes in communities than previously available. Additionally, the MDAM increases the accuracy of large‐scale, multispecies, multiseason biodiversity monitoring.

## 2 Materials and Methods

### 2.1 MDAM basic structure

To develop the MDAM, we extended previous approaches to similar multispecies abundance problems (e.g., Chandler et al., 2013; Yamaura et al., 2012). The basic structure of the MDAM includes two hierarchical processes: a biological and observation process. The biological process estimates the true abundance of multiple species on a landscape. To allow for independent responses among species, we extended a single‐species N‐mixture model approach (i.e., no information sharing between species) to model the biological process. The observation process estimates the probability of detection using the outcome of two observers using the DDO method and the true abundance from the biological process. The MDAM accounts for imperfect observation by estimating the probability that an observer detects an individual during a survey. This is calculated from the different observation outcomes between the two observers in the DDO method. Similar to the approach in the biological process, we applied the single‐species N‐mixture approach to model the detection of each species. However, we have included the multispecies N‐mixture extensions in the R code provided in Data S1. The structure of each hierarchical process within the MDAM is described below.

#### 2.1.1 Modeling abundance

We considered the likelihood for the latent abundance of species *i* at plot *j* (*N*_*ij*_) to be a function of a Poisson random variable with mean abundance per plot (λ_*ij*_) (eq. (1)). We used a Poisson distribution because we assumed that individuals and species of interest were randomly distributed across plots (Royle, 2004). To account for over‐dispersion in abundance, we modeled mean abundance of species as a random effect that varied by species (eq. (2)). We used a vague normal distribution *N* (0, 1,000) for the prior distribution (hereafter “prior”) of mean abundance.

(1) $$N_{ij}∼\text{poisson}\;\left(\lambda_{ij}\right)$$

(2) $$\lambda_{ij}∼\text{exp}\;(\log\;\cdot \;n_{ij})$$

#### 2.1.2 Modeling observations

The DDO survey method produces observations with three possible outcomes: (1) The primary observer detects an individual; (2) the secondary observer detects an individual that the primary observer misses; and (3) both the primary and secondary observer fail to detect an individual. Each of these outcomes has a different probability of occurring because they are based on a combination of events resulting from two observers. Outcome 1 is based only on the primary observer's ability to detect an individual (*p*_1_). Outcome 2 is a product of the probability that the primary observer did not detect an individual (1−*p*_1_) and the secondary observer's ability to detect an individual (*p*_2_). Outcome 3 is a product of neither observer detecting an individual (1−*p*_1_)*(1−*p*_2_). Because this process has multiple outcomes with multiple probabilities, it is a multinomial process. We modeled the observed abundance of species *i* at plot *j* at survey replicate *k* (*y*_*ijk*_) as a multinomial random variable that is a function of latent abundance (*N*_*ij*_) (eq. (1)), and three multinomial cell probabilities π_*ijk*_ that represent the DDO survey outcomes (eq. (3); Data S2).

(3) $$y_{ijk}∼\text{Multinomial}\;\left(N_{ij},\;\pi_{ijk}\right)$$

### 2.2 Simulation study

We simulated data to assess the performance of the MDAM. We used a random Poisson distribution to model true abundance for four hypothetical species randomly distributed across 20 plots. Count data were generated using a random multinomial distribution with the three cell probabilities that corresponded to the outcomes of the DDO process, described above. The count data reflected two observers using the DDO method on three replicate visits at each of the 20 plots over a single season. Detection was held constant at .3 for the primary observer and .5 for the secondary observer. We considered differences in individual observer effect as the only source of variation in detectability in the observation process. We assumed that all four species were available and observed on each plot during each survey replicate. All analyses were conducted in program R (version 3.2.0; R Development Core Team 2015) and JAGS (http://mcmc-jags.sourceforge.net). Code to generate simulated data executes the MDAM are included in Data S1.

#### 2.2.1 Assessing MDAM performance

We ran MCMC with three Markov chains for each data set. Each chain consisted of 50,000 iterations, of which the first 5,000 iterations were discarded as a burn‐in. We specified over‐dispersed starting values for three Markov chains based on recommendations from King, Morgan, Gimenez, and Brooks (2010). To assess chain convergence, we used two diagnostics: (1) Trace plots, which show all of the values of the Markov chains during the 50,000 iterations, to visually inspect chain mixing (King et al., 2010); and (2) the $\hat{R}$ statistic, an estimate of the ratio of the among‐chain variance to the within‐chain variance (Brooks & Gelman, 1998).

We used simulated data to examine the precision and accuracy of the MDAM. We compared true abundance and detection values we generated to the MDAM estimates of abundance and detection to measure precision and determine whether the abundance values from the simulations contained the true abundance values. To assess the ability of the model to recover truth, we measured coverage, or the percent of time the 95% credible interval (CRI) of the MDAM estimates of abundance and detection included the known true values of abundance and detection. We measured accuracy by calculating the mean absolute percent error of the MDAM parameter estimates for abundance and detection. Mean absolute percent error was calculated as the absolute value of the difference between the true parameter value and MDAM parameter estimates divided by the true parameter value, all multiplied by 100. To ensure that the MDAM could accurately predict parameters under a wide range of possible survey outcomes, we ran the MDAM 100 times with different starting values each time. We summarized the results of the 100 simulations to assess overall MDAM performance.

### 2.3 MDAM extension: application example

#### 2.3.1 Applying the MDAM extension

**Figure 1:**
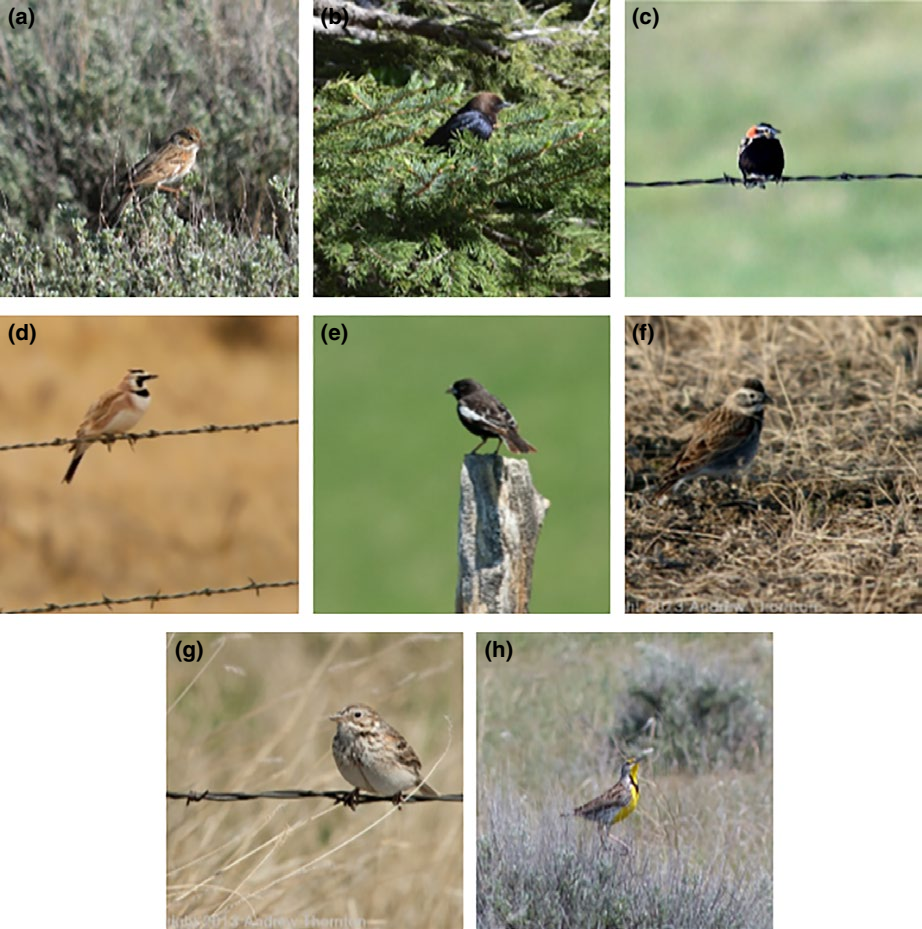
The eight avian species used in the case study data: *(*a) Brewer's sparrow (*Spizella breweri*); *(*b) brown‐headed cowbird (*Molothrus ater*); *(*c) chestnut‐collared longspur (*Calcarius ornatus*); *(*d) horned lark (*Eremophila alpestris*); *(*e) lark bunting (*Calamospiza melanocorys*); *(*f) McCown's longspur (*Rhynchophanes mccownii*); *(*g) vesper sparrow (*Pooecetes gramineus*); and *(*h) western meadowlark (*Sturnella neglecta*)

To demonstrate the applicability of MDAM extensions, we applied the MDAM to a two‐year case study using eight avian species of prairie songbird communities in eastern Montana. We selected the eight species that represent a wide spectrum of vegetation use present in sagebrush ecosystems. They range from the following: Species dependent entirely on sagebrush, Brewer's sparrow (*Spizella breweri*); to species dependent entirely on grassland vegetation, chestnut‐collared longspur (*Calcarius ornatus*), horned lark (*Eremophila alpestris*), lark bunting (*Calamospiza melanocorys*), McCown's longspur (*Rhynchophanes mccownii*), vesper sparrow (*Pooecetes gramineus*), and western meadowlark (*Sturnella neglecta*); to a species dependent on both sagebrush and grassland vegetation, the brown‐headed cowbird (*Molothrus ater*) (Figure 1). The structure of the MDAM extension is described below.

#### 2.3.2 Case study data set

**Figure 2:**
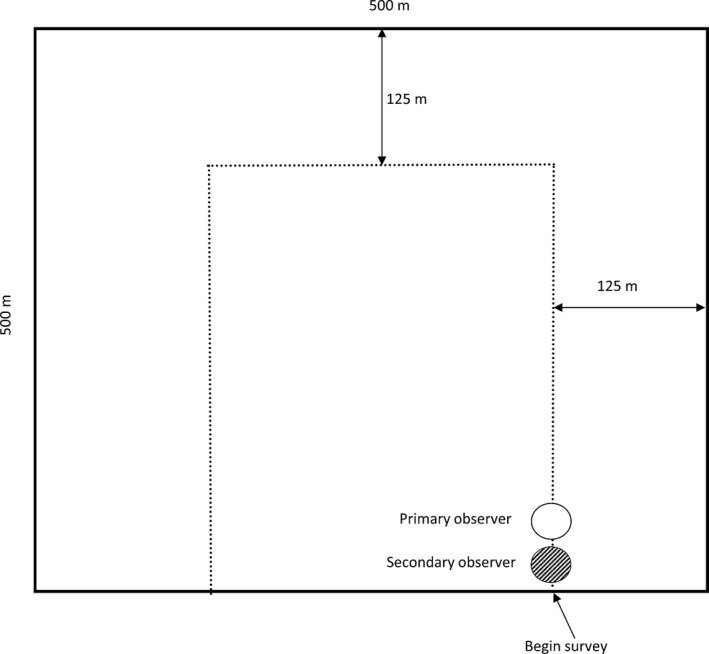
Dependent double‐observer method. The primary (open circle) and secondary observer (dashed circle) walk single‐file along the transect (dotted line) within a 500 m × 500 m sampling plot. Observers survey up to 125 m on either side of the transect. All surveys start at the lower right corner of the transect. Bold arrows indicate direction of travel

Observers conducted counts of the eight sagebrush songbird species described above using the DDO method during the peak songbird breeding season (May through July) in 2013 and 2014. The surveys were conducted on approximately 2,000 ha of private and public rangelands in Golden Valley and Musselshell counties, Montana, USA. The area is dominated by sagebrush (*Artemsia tridentata* spp. *wyomingensis*) and native grassland. Sampling plots were 25 ha (Figure 2) with 40 plots on private land and 40 plots on public land for a total of 80 plots. The plot size was based on covering 125 m from a survey transect (Figure 2) based on ≥95% of songbird detections are within 125 m of a single observer (Ralph et al. 1995). Observers surveyed each plot three times (approximately once a month in May, June, and July) over the breeding season within a year. Surveys were conducted between approximately 0600 and 1100 hrs. Surveys were not conducted during inclement weather or when winds were greater than 15 mph.

#### 2.3.3 Modeling abundance

True abundance of species *i* at plot *j* in year *y* (*N*_*ijy*_), was modeled as a Poisson random variable with mean species abundance per plot in each year (λ_*ijy*_) (eq. (4)). We included land ownership as a binary categorical covariate to account variation in the abundance of these eight species. Differences in land management associated with land ownership have been suggested to change the potential of a landscape to support biological communities (Scott et al., 2001). We let the effect of land ownership vary by species *i* to capture the variation in species' responses to land‐use practices or other variables associated with ownership. We used a log link function to relate land ownership to abundance using a linear predictor of mean species abundance per plot in each year (λ_*ijk*_). We modeled the mean species abundance per plot in each year as a function of the linear combination of a species‐specific intercept (β_0*i*_), plus a fixed effect of land ownership that varied by species (β_1*i*_), a fixed effect for year that varied by species (β_2*i*_), plus a random effect for plot (α_*j*_) to account for variation not otherwise explained (eq. (5)).

(4) $$N_{ijy}∼\text{poisson}\;\left(\lambda_{ijy}\right)$$

(5) $$\log\left(\lambda_{\mathrm{ijy}}\right)=\beta_{0i}+\beta_{1i}\times \text{land ownership}+\beta_{2i}\times \text{year}+\alpha_{j}$$

We used vague normal distributions N (0, 1,000) for the prior of the coefficients of the linear predictor of the mean species abundance per plot in each year (λ_*ijy*_). We included year as a binary categorical covariate to account for variation in abundance between years. For the random effect (α_*j*_), we used a uniform distribution ranging from 0 to 100 for the prior on the dispersion parameter.

#### 2.3.4 Modeling observations

We used the basic MDAM structure to model observations for the case study data. We modeled the observed abundance of species *i* at plot *j* in year *y* at survey replicate *k* (*y*_*ijky*_) as a multinomial random variable that is a function of true abundance (*N*_*ijy*_) (eq. (3)) and cell probabilities (π_*ijk*_) based on the DDO surveys described above (eq. (6); Data S3).

(6) $$y_{ijky}∼\text{Multinomial}\;\left(N_{ijy},\;\pi_{ijk}\right)$$

We accounted for variation in the observation process by including both individual observer and species effects. We did not include additional explanatory covariates in the observation process because additional sources of variation were reduced using timing and weather restrictions for all DDO surveys, described in *Case Study Data Set* below. We used vague normal distributions N (0, 10,000) for the priors of detectability for each observer that informed the multinomial cell probabilities.

#### 2.3.5 MDAM extension performance

We ran MCMC with three Markov chains for each data set. Each chain consisted of 50,000 iterations, of which the first 5,000 iterations were discarded as a burn‐in (see Data S1 for code). We used visual inspection trace plots (King et al., 2010) and the $\hat{R}$ statistic (Brooks & Gelman, 1998) to examine parameter convergence. We also examined the posterior density distributions, probability distributions that represent a parameter estimate, to check for smooth, unimodal posterior distributions. A unimodal posterior distribution indicates that a single, predicted value of a parameter (the parameter estimate where the peak of the distribution occurs) has the highest probability of support.

## 3 Results

### 3.1 Basic MDAM

Markov chain convergence appeared to have been reached for abundance and detection by the 5,000 iteration burn‐in period. Good convergence is represented by chains with considerable overlap, so that all chains appear almost indistinguishable from one another. In addition, all $\hat{R}$ values were near one (<1.01). Values of $\hat{R}$ close to 1 indicate that the Markov chains have converged on the single posterior value. Mean coverage across 100 repeated simulations was 0.945, or 94.5%, meaning that on average 94.5% of abundance estimates in any given simulation had a 95% CRI which included truth. Coverage for detection was similarly high at 0.925. The MDAM also provided accurate estimates. The majority of abundance estimates from the simulations (92.2%) had a mean absolute percent error between 0% and 20% (Table 2), with an overall mean value of 7.7%. The mean absolute percent errors for detection estimates were less than or equal to 5% (Table 3), with an overall mean value of 1.2%.

**Table 2:** Mean absolute percent error for abundance estimates from the multispecies dependent double‐observer abundance model. Data were simulated 100 times for four species surveyed on 20 plots three times over a season by two observers

Mean absolute percent error	% of simulations^a^
0–20	92.2
21–40	5.7
41–60	1.1
61–80	0.5
81–100	0.2
>100	0.3

a % of simulations represents the percent of simulations of 100 that fall within the given range.

**Table 3:** Mean absolute percent error for detection probability estimates from the multispecies dependent double‐observer abundance model. Data were simulated 100 times for four species surveyed on 20 plots three times over a season by two observers

Mean absolute percent error	% of simulations^a^
1	51.0
2	30.5
3	14.5
4	3.5
5	0.5

a % of simulations represents the percent of simulations of 100 that fall within the given range.

### 3.2 MDAM extension

**Figure 3:**
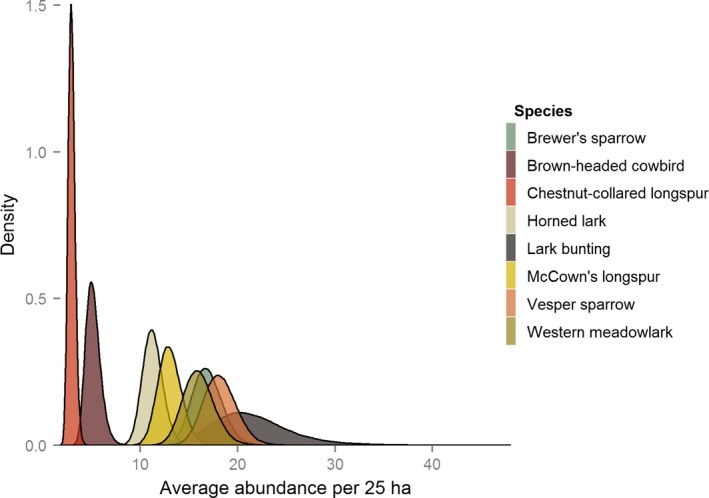
The posterior distributions of average abundance per 25 ha in 2013 on public land for eight avian species. Estimates are derived using the multispecies‐dependent double‐observer abundance model and data collected near Roundup, Montana in 2013

Markov chain convergence appeared to have been reached for all parameters: abundance, detection, and effect of private land. All $\hat{R}$ values were near one (<1.01). Posterior distributions were smooth and unimodal, suggesting convergence and adequate mixing. Figure 3 shows the posterior density distributions of the abundance estimates for the eight avian species that were analyzed with the MDAM.

The case study data consisted of 11,267 observations in 2013 and 12,175 observations in 2014 of the eight sagebrush songbird species (Table 4). In both 2013 and 2014, total observations were higher on private land (6,080 and 6,878, respectively) than public land (5,187 and 5,297, respectively), although this pattern differed by species. In 2013 and 2014, observers recorded more Brewer's sparrows, brown‐headed cowbird, lark bunting, vesper sparrow, and western meadowlark on public land than on private land. In contrast, in 2013 and 2014, there were more horned larks and McCown's longspurs observed on private land than public land. The observed number of chestnut‐collared longspurs was similar between land ownership and years.

**Table 4:** Summary of observations of eight sagebrush songbirds surveyed using the dependent double‐observer method in 2013 and 2014 near Roundup, MT. Plots refers to the number of plots of 40 in which the species was detected. Observed refers to the total number of individuals observed during the three sampling occasions

Common name	2013				2014			
	Public		Private		Public Land		Private	
	Plots	Observed	Plots	Observed	Plots	Observed	Plots	Observed
Brewer's sparrow	35	979	27	804	33	1,101	24	927
Brown‐headed cowbird	30	200	17	90	26	203	20	120
Chestnut‐collared longspur	16	168	19	272	5	209	16	197
Horned lark	33	597	37	1,015	31	870	37	1,075
Lark bunting	17	345	17	113	19	352	20	234
McCown's longspur	18	1,037	31	2,450	15	726	29	2,824
Vesper sparrow	39	1,066	39	936	38	1,057	37	1,030
Western meadowlark	40	795	40	400	40	779	39	471
Totals		5,187		6,080		5,297		6,878

**Figure 4:**
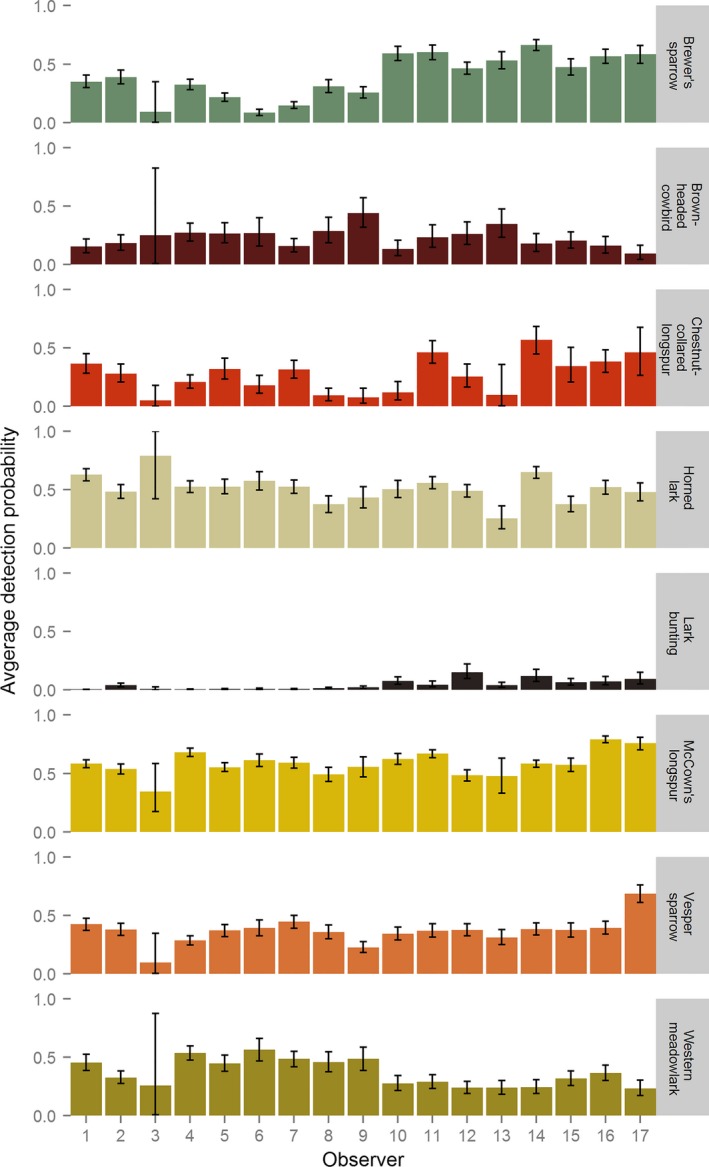
The average probability (right *y*‐axis) that an individual observer (*x*‐axis) detected each avian species (left *y*‐axis) during dependent double‐observer surveys conducted on public and private lands near Roundup, Montana, in 2013 and 2014. Black bars represent the 95% Bayesian credible intervals of the estimate

Detection probabilities varied greatly between observers and species, ranging from 0 to 0.79 (Figure 4). Lark buntings had the lowest average detection probability (0.05), followed by brown‐headed cowbird (0.23), chestnut‐collared longspur (0.27), vesper sparrow (0.37), western meadowlark (0.73), Brewer's sparrow (0.39), horned lark (0.51), and McCown's longspur (0.58).

**Figure 5:**
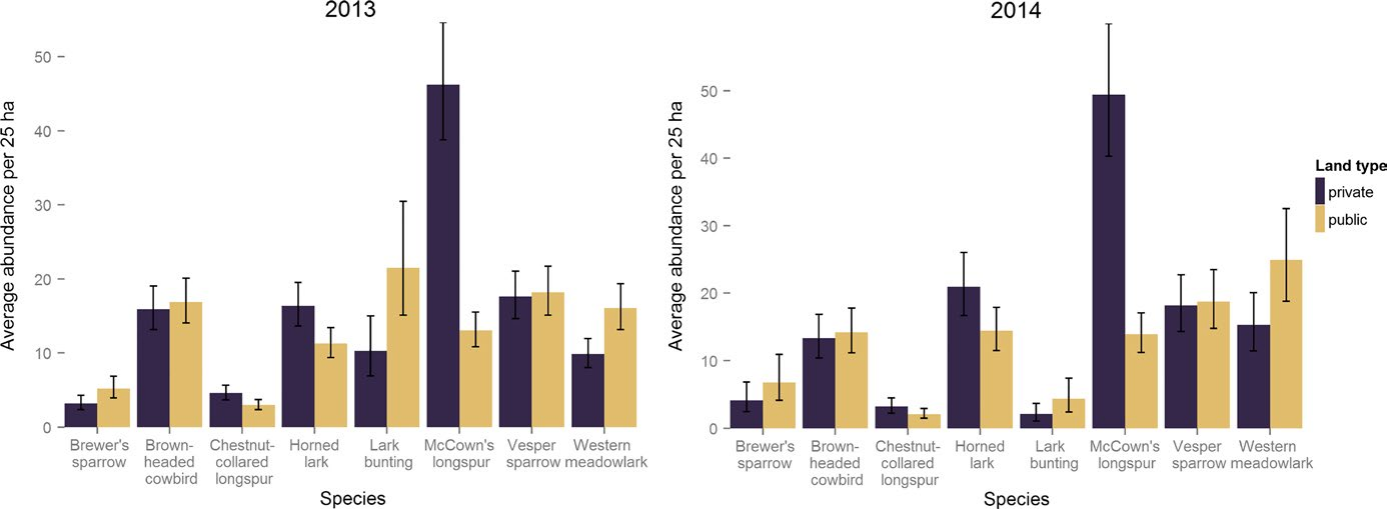
The average estimated abundance per 25 ha on public and private land for eight avian species. Black bars represent the 95% Bayesian credible intervals of the estimate. Predictions are derived from the multispecies‐dependent double‐observer abundance model using data collected near Roundup, Montana in 2013 and 2014

Predicted abundance patterns were similar for most species 2013 and 2014 (Figure 5; Table 5). There were significantly more (i.e., 95% CRIs did not overlap) individuals predicted on public land in 2013 for lark bunting and western meadowlark than private land. However, this pattern did not remain in 2014, when the difference in abundance for both lark bunting and western meadowlark was not significant between public and private lands. On the other hand, there were significantly more McCown's longspurs per 25 ha predicted on private land in 2013 and 2014 than public land. For all other species, brown‐headed cowbird, Brewer's sparrow, chestnut‐collared longspur, horned lark, and vesper sparrow, there was no significant difference in 2013 and 2014 between public land and private land (Table 5).

**Table 5:** The average estimated abundance ($\hat{N}$) and 95 percent credible intervals (CRI) per 25 ha on public and private land for eight avian species. Predictions are derived from the multispecies dependent double‐observer abundance model using data collected near Roundup, Montana in 2013 and 2014

Common Name	2013				2014			
	Public		Private		Public Land		Private	
	$\hat{N}$	95% CRI	$\hat{N}$	95% CRI	$\hat{N}$	95% CRI	$\hat{N}$	95% CRI
Brewer's sparrow	16.9	14.0–20.1	15.9	13.2–19.0	14.2	11.2–17.8	13.4	10.4–16.9
Brown‐headed cowbird	5.2	3.9–6.9	3.2	2.3–4.3	6.8	4.1–11.0	4.2	2.5–6.9
Chestnut‐collared longspur	3.0	2.3–3.7	4.6	3.7–5.7	2.1	1.4–2.9	3.2	2.2–4.5
Horned lark	11.3	9.4–13.4	16.4	13.6–19.5	14.4	11.5–17.9	20.9	16.7–26.0
Lark bunting	21.5	15.1–30.8	10.2	6.9–15.0	4.4	2.4–7.3	2.1	1.1–3.6
McCown's longspur	13.0	10.8–15.5	46.2	38.8–54.6	13.9	11.2–17.1	49.4	40.3–59.9
Vesper sparrow	18.2	15.1–21.7	17.6	14.6–21.0	18.7	14.8–23.4	18.1	14.3–22.7
Western meadowlark	16.0	13.1–19.4	9.8	8.0–12.0	24.9	18.8–32.6	15.3	11.5–20.1

**Figure 6:**
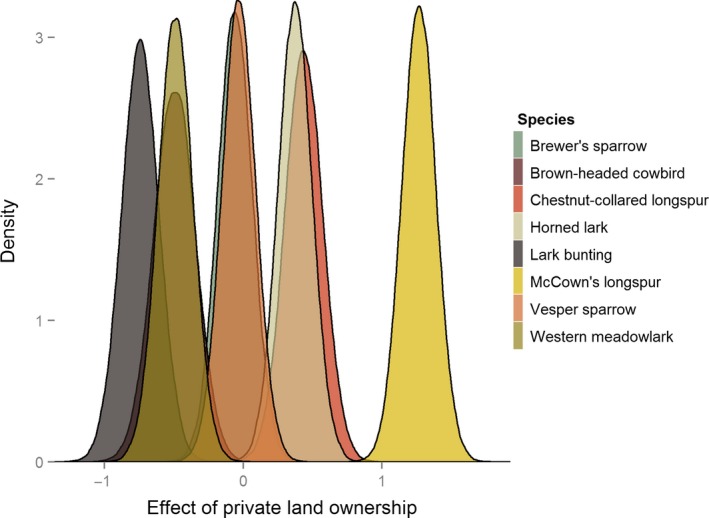
The effect of private land ownership on average abundance per 25 ha compared to public land ownership for eight songbird species on public and private lands near Roundup, Montana, in 2013 and 2014. The effect values are on the link scale

Land ownership had positive, negative, and neutral effects on the eight species examined (Figure 6). The results in the remainder of this section are presented as an estimate from the MDAM (on the link scale) and a 95% CRI in brackets. There was no significant effect (i.e., the 95% CRI overlapped with 0 and the most support in posterior distribution was for values at or near 0 on the link scale) on the estimated abundance for two of the eight species examined: Brewer's sparrow (−0.05 [−0.30–0.18]) and vesper sparrow (−0.03 [−0.27–0.21]). Private land ownership had a significant positive effect on the predicted abundance of chestnut‐collared longspur (0.43 [0.16–0.70]), horned lark (0.37 [−0.13–0.62]), and McCown's longspur (1.26 [1.02–1.51]). Private land ownership had a significant negative effect on three species: brown‐headed cowbird (−0.49 [−0.79 to −0.20]), lark bunting (−0.74 [−1.01 to −0.48]), and western meadowlark (−0.49 [−0.73 to −0.23]).

## 4 Discussion

The MDAM extends previous multispecies N‐mixture models to include a removal‐based survey method that reduces the rate of false positives. It provides flexibility for synthesizing multiple sources of data that are hindered by imperfect detection from biological (e.g., differences in abundance that arise from different land use) and observation process (e.g., observer performance). Although it is similar to the multinomial abundance model published by Kéry and Royle (2010) and the model published by Chandler et al. (2013), it is the first to implement the DDO methodology in the multispecies N‐mixture structure.

All performance diagnostics indicated that the MDAM is an accurate and suitable model for multispecies analyses. Information from the DDO method provided detailed encounter history information for each detected individual that was used to model the observation process. With the MDAM extension, each encounter history incorporated individual observer effects and species effects. The MDAM consistently predicted precise values that contained the true parameters when run 100 times with different starting values, indicating that the predications are reliable. Similarly, the convergence diagnostics and posterior distributions of the MDAM extension indicated that the MDAM extension converged well on posterior distribution estimates.

The predictions of the MDAM extension were biologically sound and congruent with other studies. The community composition of this prairie system predicted by the MDAM extension is similar to songbird communities in nearby sagebrush and mixed‐grass communities (Bradford et al., 1998; Jones, Scott Dieni, & Gouse, 2010; Reinkensmeyer, Miller, Anthony, & Marr, 2007). The most abundant species, McCown's longspur, western meadowlark, vesper sparrow, and Brewer's sparrow, were consistent with other findings (Bradford et al., 1998; Jones et al., 2010). We found that land ownership had a neutral or positive effect on predicted abundance for the majority of species, five of eight, which we investigated. The positive effect of private land on chestnut‐collared longspur, horned lark, and McCown's longspur abundance was consistent with other findings about private lands, which often support more species than public or protected lands (Scott et al., 2001).

The MDAM provides many benefits that result from both the MDAM model structure and the DDO survey method. The MDAM structure does not require replication at some sample plots like other multispecies N‐mixture models because of the detectability information contained within the DDO observations. Therefore, it is possible that field efforts could be reduced with similar information yield, which is useful when trying to allocate limited personnel and financial resources. Using the DDO method, observers can work together to identify a bird and ensure double counting is not occurring, likely reducing false positives. The observation outcomes, primary observer detects an individual or secondary observer detects an individual that the primary missed, must be correctly recorded. Once that occurs, the observers have flexibility to collaborate to identify characteristics of the individuals (e.g., species, sex). The ability of the observers to work together on identification, with the stipulation that the observation outcome has to be correctly recorded, also allows new observers to be quickly trained in bird identification.

The MDAM structure is generalizable and can be applied to many different systems to estimate multispecies abundance. The multispecies abundance data from the MDAM can be used to derive abundance‐based biodiversity metrics that summarize species richness and evenness, or relative abundance to other species. In addition, it is possible to relax many of the assumptions of the MDAM. For example, the assumption that every species is available for sampling during the observation process is unrealistic (Dorazio & Royle, 2005). However, this can be addressed in the MDAM by adding another level to the hierarchical model that represents the animal's availability, as described by Kéry and Schaub (2012). In our case study example, the only explanatory covariate for latent abundance is land ownership. The MDAM can accommodate additional biotic or abiotic covariates that are plot‐ and/or ecosystem‐specific to explain variation in abundance. In addition, abundance estimates from the MDAM can be used in an integrated population model (Kéry & Schaub, 2012). This can provide a clearer picture of the mechanisms driving changes in abundance and biodiversity. The MDAM can also be used to concurrently track the abundance of a single species and a biodiversity parameter of interest. If monitored over multiple seasons, this can provide a potential method to determine whether a focal species reliably tracks changes in a community. Finally, although the DDO was developed as a bird survey method (Nichols et al., 2000), it can be used on additional taxa. Double‐observer methods have already been used for marine and terrestrial mammals (Buckland, Laake, & Borchers, 2010; Griffin et al., 2013; Hoef, Cameron, Boveng, London, & Moreland, 2014) and amphibians (Becker, Moorman, DePerno, & Simons, 2013).

The patterns of abundance of multiple species are fundamental to understanding biodiversity. The MDAM provides a framework of reliable multispecies abundance predictions and can accommodate extensions that have important implications for conservation. The MDAM has the flexibility to incorporate long‐term, large‐scale, and multitaxa data. It can provide data‐driven solutions to reduce cost and effort put into biodiversity monitoring while still providing accurate, high‐resolution data. In addition, there may be further extensions of the MDAM, such as methods to quantify the rates of false positives that would allow for an unprecedented accuracy in multispecies monitoring. Given the field and data benefits of the MDAM and its ability to accommodate extensions, the MDAM can be an instrumental tool for the future of biodiversity conservation.

## Data Accessibility

R scripts: Data are uploaded online as Supporting Information.

## Author Contributions

JG and VJD conceived the ideas and designed field methodology; JG and VJD collected the data; JN and JG designed the model and analyzed the data; JG led the writing of the manuscript. All authors contributed critically to the drafts and gave final approval for publication.

## Conflict of Interest

None declared.
