# Supplementary material for: A multispecies dependent double‐observer model: A new method for estimating multispecies abundance
Source: Ecol Evol. 2017 Apr 4;7(10):3425–35. doi: 10.1002/ece3.2946 (PMC5433993; doi:10.1002/ece3.2946)
Supplement: Supplementary file 1 [file ECE3-7-3425-s001.docx]

## Supporting Information - A

### Basic MDAM ###

# Create function (sim.fun) to simulate abundance data for four species sampled

# at 20 sites using dependent double-observer method with three repeated visits

# over a single survey season (year)

# Need JAGS 4.0, and the following packages: R2jags, rjags, and coda.

sim.fun <- function(n.sites){

## Setup the logistics of sampling

# Number of sites

n.sites <<- n.sites

# Number of visits to each site

n.reps <- 3

# Number of observers

n.observers <- 2

# Number of sp

n.sp <- 4

# Number of observations

n.obs <- n.sites * n.reps *n.sp

# Indices for long format

# prim = primary observer

#sec = secondary observer

prim <- sample(1:2, n.sites*n.reps*n.sp, replace = T)

sec <- ifelse(prim == 1, 2, 1)

# A quick check that it worked

all((prim + sec) == 3)

# Generate site information

site <- rep(1:n.sites, each = n.reps*n.sp)

# Generate survey replicate information

reps <- rep(rep(1:n.reps, n.sites), n.sp)

# Generate species information

sp <-rep(1:n.sp, each = n.sites*n.reps)

# Detection probability of primary observer

P <- vector("numeric")

P[1] <- 0.3

# Detection probability of secondary observer

P[2] <- 0.5

# Sum of P's should be less than 1, where the remainder represents the

# proportion of the sampled population not observed

stopifnot((P[1] + P[2]) < 1)

cat("\nProbability of not capturing birds", 1 - (P[1] + (P[2] * (1 - P[1]))),

"\n\n")

## Biological parameters

# Mean abundance across sites, one for each species

lambda <- c(20, 150, 300, 1000)

# Proportion of the population captured at each session

p.cap <- P[1] + (P[2] * (1 - P[1]))

# Proportion of population not captured at each session

p.nocap <- 1 - p.cap

## Simulation

# Initialize matrix (N) to hold values of abundance corrected for availability

N <- array(NA, dim = c(n.sites, n.reps, n.sp))

# Initialize matrices to hold values of observations and probability of detection

#Columns are outcomes of the multinomial

y <- cp <- matrix(NA, nrow = n.obs, ncol = n.observers)

# Initialize matrix (M) to hold values of true abundance

M <- matrix(NA, n.sites, n.sp)

for(i in 1:n.sites){

M[i,] <- rpois(n.sp, lambda)

}

# Abundance corrected for availability during each survey replicate

for(i in 1:n.sites){

for(j in 1:n.reps){

for(k in 1:n.sp){

N[i,j,k] <- rbinom(1, M[i,k], p.cap)

}

}

}

# Number observed

for(i in 1:n.obs){

cp[i,] <- c(P[prim[i]], P[sec[i]] * (1 - P[prim[i]]))

y[i,] <- c(rmultinom(1, N[site[i], reps[i], sp[i]], cp[i,]))

}

#Put the data together in long format

input <- data.frame(cbind(y[,1:2], y[,1]+y[,2], site, reps, sp, prim, sec))

colnames(input)[1:3] <- c("y1", "y2", "ncap")

##############################################################################

## Basic MDAM ##

# JAGS model to estimate parameters

sink("MDAM_sim.txt")

cat("

model{

# Priors

# Linear predictor on abundance, setup for species variation only,

# abundance assumed the same at every site

for(i in 1:n.sp){

log.n[i] ~ dnorm(0, 0.001)

mu.lambda[i] <- exp(log.n[i])

}

# Population size of each species at each site

for(i in 1:n.sites){

for(k in 1:n.sp){

N[i,k] ~ dpois(mu.lambda[k])

}

}

# Individual observer detection probability, no variation

for(i in 1:n.observers){

p[i] ~ dbeta(1, 1)

}

# Likelihood

for(i in 1:n.obs){

# Indices always follow site, reps, species order

# Capture probabilities

# Seen by observer #1

cp[i,1] <- p[prim[i]]

# Seen by observer #2 and not seen by observer #1

cp[i,2] <- p[sec[i]] * (1 - p[prim[i]])

# Seen by somebody

pcap[i] <- sum(cp[i,])

# Not seen by either observer

pnocap[i] <- 1 - pcap[i]

# Adjust the probability of capture to the prop available

# 2 is for number of outcomes (probabilities for obs1 and obs2)

for(j in 1:2){

muc[i,j] <- cp[i,j]/pcap[i]

}

# Realizations

# Number captured (ncap) and population size (N)

ncap[i] ~ dbin(pcap[i], round(N[site[i],sp[i]]))

y[i,] ~ dmulti(cp[i,1:2], ncap[i])

}

}

", fill = T)

sink()

##############################################################################

# Format JAGS data

data <- list("y" = input[,1:2],

"prim" = input$prim,

"sec" = input$sec,

"n.obs" = nrow(input),

"n.observers" = n.observers,

"n.sites" = length(unique(input$site)),

"site" = input$site,

"n.sp"=length(unique(input$sp)),

"ncap" = input$ncap,

"sp"=input$sp)

# R2jags requires the data is in the global environment. Because this is in a

# function need to write it to the global environment each time

list2env(data, envir=globalenv())

require(R2jags)

# Set initial values (inits)

inits <- function(){list(

log.n = log(lambda),

p = c(0.3, 0.5),

N = M*2 )}

# Define parameters for the MDAM to track (parms)

parms <- c("p", "N", "mu.lambda", "pcap")

# Save JAGS output (out) and specify initial values, parameters to track, model to run, number

# of chains to run, number of iterations to run, burn-in period, and thinning

out <- jags.parallel(data=names(data), inits, parms, "MDAM_sim.txt", 3, 50000, 1000, 1)

# Parameter to track mean absolute percent error (m) can be calculated from output using:

# m<- list("P" = round(cbind(P, out$BUGS$mean$p, 100 * abs(P - out$BUGS$mean$p)/P), 2),

# "N" = round(cbind(M, out$BUGS$mean$N, 100 * abs(M - out$BUGS$mean$N)/M), 2))

# Parameter to track coverage can be calculated from output using:

# coverage<-list("Pcov" = ifelse(P>(quantile(out$BUGS$sims.list$p,.025)) &

# P<(quantile(out$BUGS$sims.list$p,.975)), 1, 0), "Ncov" =

# ifelse(M>(quantile(out$BUGS$sims.list$N,.025)) &

# M<(quantile(out$BUGS$sims.list$N,.975)), #1, 0))

# Alternative output to view mean absolute percent error and coverage (out)

# out<-list(m,coverage)

MDAMoutput<-list(out)

}

#Create function to specify the number of times and for how many sites the sim.fun should run

sim.fun.rep<-function(n.times, n.sites){

replicate(n.times, sim.fun(n.sites), simplify = F)

}

##############################################################################

#Multispecies N-mixture model extensions

#Species information sharing in the biological process

#Replace line 113 with line 212 and add lines 213-215 between lines 111 and 112

loglam[i] ~ dnorm(mu.lam, tau.lam)

mu.lam ~ dnorm(0,0.01)

tau.lam <- 1/sd.lam^2

sd.lam ~ dunif(0, 200)

#Species information sharing in the observation process

#Replace line 122 - 125 with lines 219 - 235

tau.person ~ dunif(0, 10)

for(i in 1:n.observers){

person.eff[i] ~ dnorm(0, tau.person)

}

tau.bird ~ dunif(0, 10)

for(i in 1:n.sp){

bird.eff[i] ~ dnorm(0, tau.bird)

}

p.int ~ dnorm(0, 0.001)

for(i in 1:n.observers){

for (j in 1:n.sp){

logit(p[i,j]) <- p.int + bird.eff[j] + person.eff[i]

}

}

## Supporting Information - B

The likelihood function for the observation of a single species using the basic MDAM.

$$L\left( p_{1},p_{2}\left| {\{y}_{1i},y_{2i}, N_{i}-(y_{1i}+y_{2i}) \right\}, N \right)=$$

$$\prod_{i=1}^{obs} \left[ \binom{N}{y_{1i}, y_{2i}, N_{i}-{(y}_{1i}+y_{2i})}({p_{1})}^{y_{1i}}\left( p_{2}\left( 1-p_{1} \right) \right)^{y_{2i}} {{(1-(p}_{1}+p_{2}(1-p_{1})))}^{N_{i}-{(y}_{1i}+y_{2i})} \right]$$

$p_{1}$ = probability of detection of the primary observer

$p_{2}$ = probability of detection of the secondary observer

$y_{1i}$ = count of individuals by the primary observer during observation *i*

$y_{2i}$ = count of individuals by the secondary observer during observation *i*

$N_{i}$ = total number individuals available for detection during observation *i*

$N$ = total number individuals available for detection

$obs$ = observation

## Supporting Information - C

The likelihood function for the observation of a single species using the MDAM extension.

$$L\left( pcap, p_{1}, p_{2} | y_{1i}, C_{i}, N \right)=$$

$$\prod_{i=1}^{obs} \left[ \binom{N}{C_{i}}({pcap)}^{C_{i}}\left( 1-pcap \right)^{N-C_{i}}\binom{C_{i}}{y_{1i}}{{(p}_{1})}^{y_{i}}\left( \left( 1-p_{1} \right)p_{2} \right)^{C_{i}-y_{1i}} \right]$$

$pcap$= probability that at least one observer detects an individual

$p_{1}$ = probability of detection of the primary observer

$p_{2}$ = probability of detection of the secondary observer

$y_{1i}$ = count of individuals by the primary observer during observation *i*

$C_{i}$ = total number individuals detected by either the primary or secondary observer during observation *i*

$N$ = total number individuals available for detection

$obs$ = observation
